# Infection prevention in medical education – results of a descriptive cross-sectional study in Germany

**DOI:** 10.3205/zma001659

**Published:** 2024-02-15

**Authors:** Paul-Dierk Tingelhoff, Frank Hufert, Claudia Kiessling, Bertram Otto

**Affiliations:** 1 Witten/Herdecke University, Faculty of Medicine, Education of Personal and Interpersonal Competencies in Health Care, Witten, Germany; 2 Brandenburg Medical School Theodor Fontane Senftenberg, Institute of Microbiology and Virology, Senftenberg, Germany; 3 Klinikum Ernst von Bergmann, Clinic for Gastroenterology, Hepatology, Infectiology and Rheumatology, Klinikum Potsdam, Germany

**Keywords:** medical education, infection prevention, curriculum development, examinations, serious games, gamification, innovative teaching methods, hygiene

## Abstract

**Objective:**

The aim of the study was to assess the current curricular status of content on infection prevention in hospitals during medical education prior to the development of a serious game on infection prevention in hospitals. In addition, the data collected was to be contrasted with the training for a specialist nurse in hygiene and infection prevention (FKHI).

**Methodology:**

In an online survey, persons in charge of medical degree programs and continuing education centers for FKHI, SkillsLabs and professional associations in Germany were asked to answer 28 questions on framework conditions, teaching, examinations, and gamification.

**Results:**

Data was collected for 22 medical degree programs and 5 FKHI continuing education centers. Due to the low response rate, the data for the FKHI was only analyzed in summary form. On average, 13.5 teaching units (median) are available in medical studies. Six degree programs have a longitudinal curriculum. In 7 of the 22 degree programs, teaching is based on the National Competency-Based Learning Objectives Catalogue (NKLM). Almost all locations teach this content in lectures (n=18) and/or in internships (n=13). Teaching and examinations are most common in the third year of study (n=12). In addition to practical OSCE examinations (n=5), written (n=12) and computer-based (n=8) examinations are used in particular. Gamification is known as a didactic approach to some extent but is not used for teaching infection prevention.

**Conclusions:**

Infection prevention in hospitals is given relatively low priority in medical education. Teaching and examinations are based on traditional knowledge-oriented formats, although practical teaching and practical examinations are established at some locations. In contrast to the FKHI, learning objectives currently appear to be less standardized. Further interprofessional development of teaching would be desirable in the future.

## Zusammenfassung

**Zielsetzung:**

Ziel der Studie war es, den curricularen Ist-Zustand von Inhalten zur Infektionsprävention im Krankenhaus im Medizinstudium im Vorfeld der Entwicklung eines Serious Game zur Infektionsprävention im Krankenhaus zu erheben. Zusätzlich sollten die erhobenen Daten mit der Weiterbildung zur Fachgesundheits- & Krankenpflege Hygiene und Infektionsprävention (FKHI) kontrastiert werden.

**Methodik:**

Mittels Onlinebefragung wurden Fachverantwortliche der Medizinstudiengänge und Weiterbildungsstätten für FKHI, von SkillsLabs und Fachschaften in Deutschland gebeten, 28 Fragen zu Rahmenbedingungen, Unterricht, Prüfungen sowie Gamification auszufüllen.

**Ergebnisse:**

Es wurden Daten für 22 Medizinstudiengänge und 5 FKHI-Weiterbildungsstätten erhoben. Aufgrund des geringen Rücklaufs wurden die Daten für die FKHI nur zusammenfassend ausgewertet. Im Medizinstudium stehen im Durchschnitt 13,5 Unterrichtseinheiten (Median) zur Verfügung. Sechs Studiengänge haben ein longitudinales Curriculum. In sieben der 22 Studiengänge orientiert sich die Lehre am Nationalen kompetenzbasierten Lernzielkatalog (NKLM). Fast alle Standorte vermitteln diese Inhalte in Vorlesungen (n=18) und/oder in Praktika (n=13). Am häufigsten wird im dritten Studienjahr unterrichtet und geprüft (n=12). Neben praktischen OSCE-Prüfungen (n=5), werden vor allem schriftliche (n=12) und computerbasierte (n=8) Klausuren eingesetzt. Gamification ist als didaktischer Ansatz zum Teil bekannt, wird jedoch nicht für den Unterricht der Infektionsprävention angewendet.

**Schlussfolgerungen:**

Der Infektionsprävention im Krankenhaus wird im Medizinstudium ein relativ geringer Stellenwert zugewiesen. Lehre und Prüfungen orientieren sich an traditionellen wissensorientierten Formaten, wobei praktischer Unterricht und praktische Prüfungen an einigen Standorten etabliert sind. Lernziele erscheinen aktuell im Gegensatz zur FKHI wenig standardisiert. Eine interprofessionelle Weiterentwicklung der Lehre wäre zukünftig anzustreben.

## 1. Introduction

### 1.1. Background 

In recent years, the covid-19 pandemic has led to increased social awareness of the importance of hygiene measures and infection prevention. Thanks to numerous information campaigns, large sections of the population are now aware of different types of masks and the importance of washing hands to prevent infectious diseases. The question arises as to what extent the increased social awareness of infection prevention issues as an interprofessional task is and should be reflected in the education, training and continuing education of healthcare professionals, particularly in medicine and nursing [1]. In line with the corresponding EU directive, medical studies comprise a total of 5,500 hours [https://www.gesetze-im-internet.de/_appro_2002/BJNR240500002.html], of which 1,920 hours are accounted for by the practical year at the end of the course. For the remaining five years of medical studies, 3,580 hours are still available, of which – as of 2010 – an average of 14 hours were spent on teaching theory and practical skills in the field of hygiene [2]. Outside of the subject of hygiene, infection prevention in hospitals is often taught as an interdisciplinary subject in medical studies, although our research has found no valid data that can show which infection prevention content is taught and tested by other specialist disciplines in hospitals. 

In comparison, training in healthcare and nursing includes a total of 120 hours of instruction on the subject of hygiene [3]. Following the conversion of training to generalist nursing training in recent years, the hours are no longer specified.

In addition to the knowledge and skills that every doctor and every nurse should have with regard to infection prevention and should therefore be reflected in undergraduate training, it is primarily the specialists in hygiene and environmental medicine and the hygiene specialists in nursing who advise and monitor the hospital in these matters. However, according to physician statistics from the German Medical Association (2019), there are only 116 specialists in hygiene and environmental medicine working in hospitals in Germany [4]. Training for a specialist nurse in hygiene and infection prevention (FKHI; specialist nurse in hygiene) qualifies participants for active hygiene and infection prevention in healthcare facilities. During the two-year training phase, participants complete several theoretical modules and a practical part in the form of a 25-week internship [5] in accordance with the concept of the framework curriculum of the German Society for Hospital Hygiene. In comparison, the importance and coverage of infection prevention and hygiene in medical education is rather low. 

With regard to the theoretical foundation in infection prevention in the context of medical studies, the current subject catalog, the basis for the state examinations and thus also trend-setting for curricular teaching in Germany, includes instruction on “Fundamentals of general, hospital and epidemic hygiene” (IMPP 2013). Based on this, the subject of hygiene always provided a reliable proportion of questions in the state examination, e.g. nine out of 320 questions in autumn 2019. However, even in the exemplary state examination, two questions on antisepsis were answered correctly by only 32% and 39% of exam participants respectively [https://next.amboss.com/de/article/WQ0PEf]. As to the performance of medical students in practical infection prevention skills, which are now frequently practiced in the skills labs, only 43% of Austrian students carry out hygienic hand disinfection in accordance with WHO guidelines [6]. A study in the DACH region with predominantly German faculties came to the conclusion that only 54% of the students surveyed rated themselves as good or very good at correct hand disinfection [7]. In addition, it was shown that both medical students and clinical staff overestimate the actual accuracy of hand hygiene, and that the workplace culture is deficient in terms of error communication [7], [8]. The authors concluded that these trainings should also be taught in conjunction with meta-competencies such as speaking-up skills and communication. Furthermore, quite a few medical students would like to be instructed in correct hand disinfection in the first semester with repeated training in each subsequent semester [7].

An interdisciplinary and interprofessional teaching approach would be desirable, particularly in view of the clinical-practical relevance of infection prevention for all medical disciplines [9], [10]. In the National Competence-Based Learning Objectives Catalog for Medicine (NKLM), which is to form the basis for teaching within undergraduate medical education throughout Germany in the coming years, learning objectives on infection prevention in hospitals are primarily found in the chapter on higher-level competencies (Chapter VIII of the NKLM), i.e. like other higher-level competencies, these are interdisciplinary. They can usually be found under other keywords and search terms, such as “hygiene”, “microbiology”, “pathogen spectrum”, “virology” and therefore no longer as purely subject-related teaching content. An interdisciplinary, competence-based approach is also aimed for in generalist nursing training [11].

So how can medical curricula be amended to place more emphasis on infection prevention in hospitals, the pertinent knowledge and application-related skills? A stronger focus on competencies in the sense of acquiring practical knowledge and skills, and thus also a greater practical relevance, is offered above all by the internships already described, in which hand disinfection is demonstrated and practiced [12]. Interprofessional training, as in other countries, would underline the cross-professional importance of infection prevention [13].

Publications and field reports seem to indicate that a lecture-centered methodology has prevailed in hygiene teaching to date, which focuses on microbiological aspects and statistics of nosocomial infections, sometimes supplemented by practical courses on hand disinfection [14], and is often perceived as “boring” by students overall [7]. Additional courses, in particular practical courses, show positive effects on knowledge retention and acceptance among students [15]. Didactic approaches that promote the acquisition of practical knowledge are less common in hygiene and infection prevention. Possible teaching formats include problem-based learning [16], [17], scenario-based learning [18] or – a relatively new development in medical education – serious games [19]. 

On the one hand, serious games have the effect of bringing people together as a gaming community and encouraging them to interact and get to know each other. On the other hand, serious games aim to motivate players to adapt their conventional behavior in challenging situations, to find new strategies for dealing with these situations and to think innovatively in the process. Through playful elements, serious games focus attention on achieving a common game goal [20]. Serious games are still rarely used in medical education and their effect on learning outcomes has been little studied [21], [22]. However, studies show that the use of serious games encourages students to learn for longer and that doctors in further training have been able to successfully build their knowledge [23]. 

### 1.2. Aim of the study

A card game called HygienX was developed at Witten/Herdecke University in collaboration with various experts from other locations to support the acquisition of patient-related application of knowledge by medical students and other healthcare professions, e.g. the nursing professions, in addition to teaching. The aim of this study was to descriptively determine which learning objectives, teaching formats and examination formats are currently used in medical studies on the topic of infection prevention and to what extent, prior to the development of the game. The results were to be contrasted descriptively with the training for a specialist nurse in hygiene and infection prevention in order to identify differences and similarities that could represent development potential for medical studies (and further training to become a hygiene specialist). 

## 2. Methods

A descriptive cross-sectional survey was conducted using an online questionnaire at all state-recognized medical faculties and universities (n=39) as well as nursing schools with a training for a specialist nurse in hygiene and infection prevention (n=25), both public and private.

### 2.1. Survey instrument

The questionnaire (FB) was developed in a multi-stage process by an interprofessional research team consisting of a nurse, a specialist representative for microbiology and virology, an expert in medical education and a doctor in further training as an internist and infectiologist. The methodology was based on the principles of empirical social research [24], [25] and previously published descriptive cross-sectional studies in the field of medical education [26], [27], [28]. An initial version was created using the open source software “Lime Survey” (version 3.28.21) and tested within this group and by an external nursing scientist. 

The final questionnaire contained a total of 28 items (see attachment 1 ). Of these, ten questions related to the organization and structure of the study or training courses, ten questions referred to the curriculum and teaching of infection prevention in hospitals, of which two questions focused on the knowledge and use of serious games, and seven questions concerned the respective examination system. A final item was used for free comments and remarks. It was important that the contact persons were as familiar as possible with the events and examinations in which the teaching of infection prevention skills plays a role.

### 2.2. Participants and recruitment

We contacted all hygiene and infection prevention representatives of the human medicine degree programs in Germany (n=39) directly in March 2022. In addition, the invitation to participate in the survey, which took place from March 2022 to April 2022 inclusive, was sent to the respective professional societies, the German Society for Hospital Hygiene (DGKH), the German Society for Microbiology and Hygiene (DGHM) and the Society for Hygiene, Environmental Medicine and Prevention (GHUP). Persons in charge of the skills labs were also asked to complete the questionnaire via an invitation to the Committee for Practical Skills of the DACH Association for Medical Education (GMA), as were the student representatives of medical students, who were invited via student representatives of the faculties of medicine in Germany and via the working group Medical Education of the Bundesvertretung der Medizinstudierenden in Deutschland (bvmd). The individual training locations for specialist further training in specialized healthcare and nursing hygiene were also contacted directly. In addition, invitations were sent to the Vereinigung Hygienefachkräfte der Bundesrepublik Deutschland e.V. (VHD).

### 2.3. Data analysis

After the survey was completed, results were exported to Microsoft Excel 2022 and analyzed descriptively. Where several questionnaires were completed at one location, two authors checked the results for consistency and plausibility. Discrepancies were discussed and corrected. Results were then merged on the basis of the plausibility check. Other possible survey errors related to the sample and non-sample-related errors were also discussed and, if necessary, corrected (e.g. missing data, measurement errors, other possibly implausible information provided by participants). All information was treated confidentially and participants were reassured that results would be reported anonymously.

### 2.4. Ethics vote

The study was reviewed by the Ethics Committee of the Medical Faculty of Witten/Herdecke University and no ethical or professional concerns were raised (UE No. 140-13).

## 3. Results

### 3.1. Response rate and description of the sample

**Figure 1:**
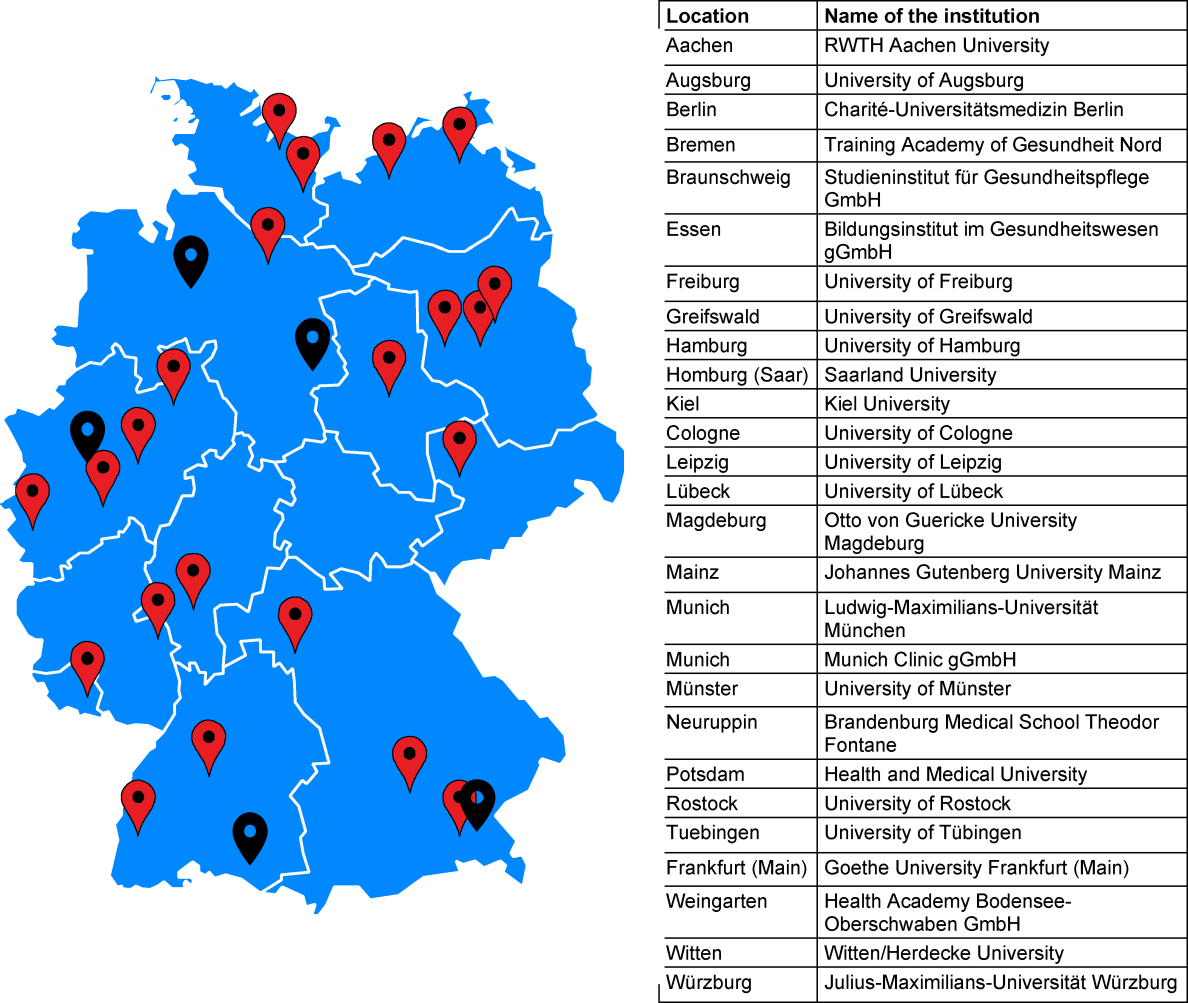
Representation of all locations participating in the survey. Note: Faculties of human medicine are shown in red, locations providing trainings for a specialist nurse in hygiene and infection prevention are shown in black

Fifty-three representatives of the contacted locations of faculties of human medicine and eight representatives of further training centers for nursing specialists in hygiene took part in the survey. After checking and merging duplicate responses, usable data sets were available for 22 medical degree programs (55%) and from 5 training centers (20%). Figure 1 (Fig. 1) provides an overview of the locations that took part in the survey. Due to the low response rate for further nursing training in hygiene, a detailed evaluation was not carried out. The most important results are summarized at the end of the results section under 3.4. 

### 3.2. Organization and implementation of teaching on infection prevention in hospitals

#### 3.2.1. Who is responsible for teaching infection prevention in hospitals?

As a rule, the specialist responsibility for infection prevention lies with chairs, institutes or professorships for hygiene and/or infection prevention and/or environmental medicine and/or microbiology and virology, in a few cases also in occupational medicine (n=1), infectiology (n=2) or hospital hygiene (n=1).

#### 3.2.2. How much is taught?

**Figure 2:**
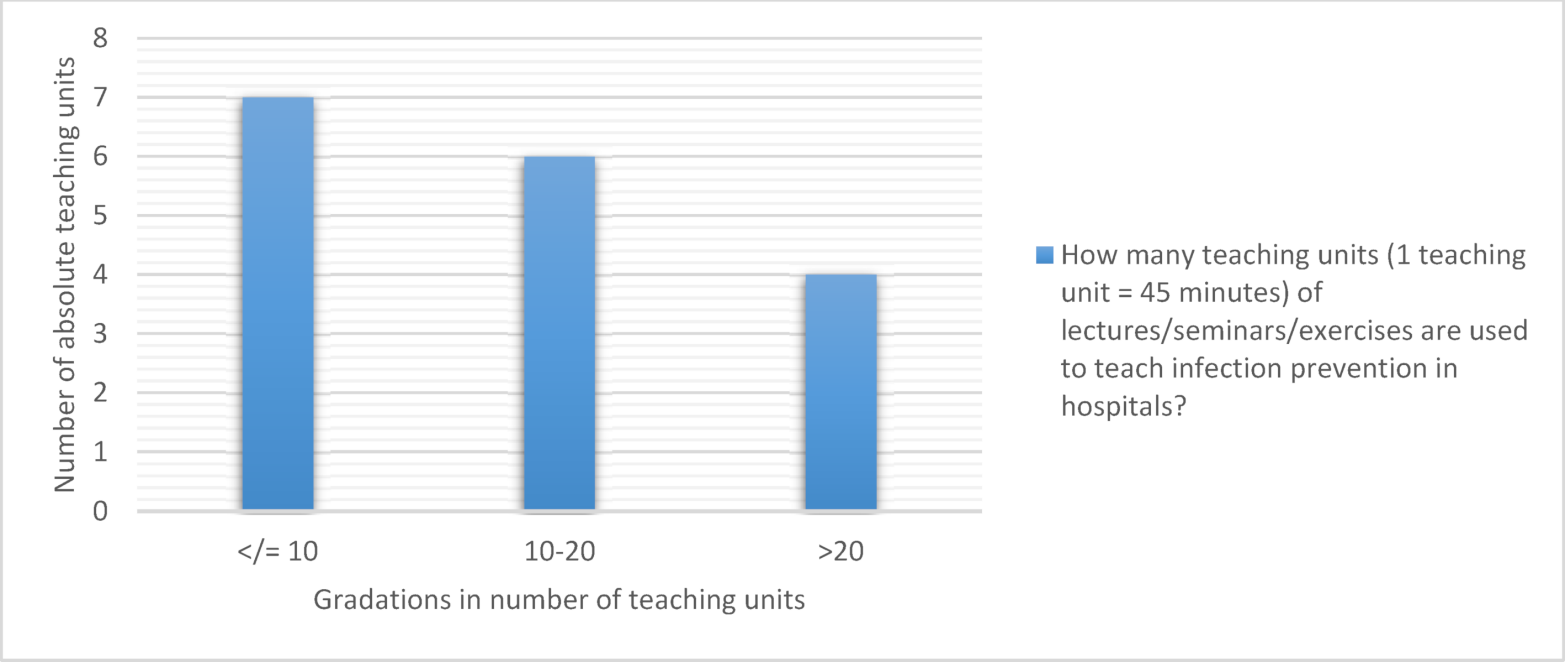
Time spent on teaching infection prevention in hospitals during medical studies (N=17; no answer was available for 5 locations)

Figure 2 (Fig. 2) provides an overview of how many teaching units (TU, 45 minutes each) are available at the medical locations in the form of lectures, seminars and/or exercises for teaching infection prevention in hospitals. The mean value of the total time spent is 19.9 teaching units (arithmetic mean) or 13.5 teaching units (median) (see figure 2 (Fig. 2)).

#### 3.2.3. When are lessons held?

**Figure 3:**
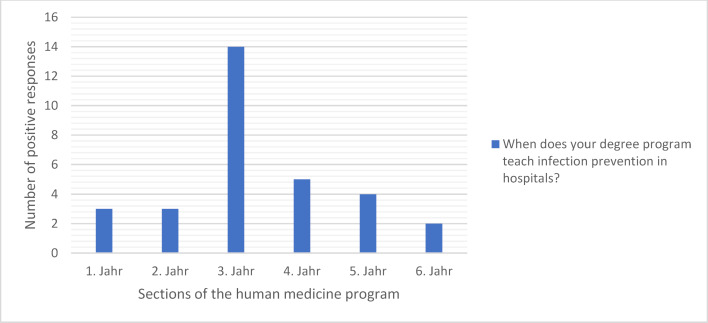
Time of teaching on infection prevention in hospitals during medical studies (Multiple answers possible; answers from 19 locations; 31 answers in total)

Teaching in this subject area is spread over at least two academic years in nine (40%) of 22 degree programs and therefore takes place on a recurring basis. In six degree programs, a teaching program exists exclusively in one of six years of study, with the focus of teaching in the third year of study (see figure 3 (Fig. 3)). 15 out of 22 faculties teach in the standard study program, in which the content examined is usually taught in the first clinical semester. At faculties with a model degree program (n=7), there is a distribution over several academic years. 

#### 3.2.4. Which teaching formats/methods are used?

**Figure 4:**
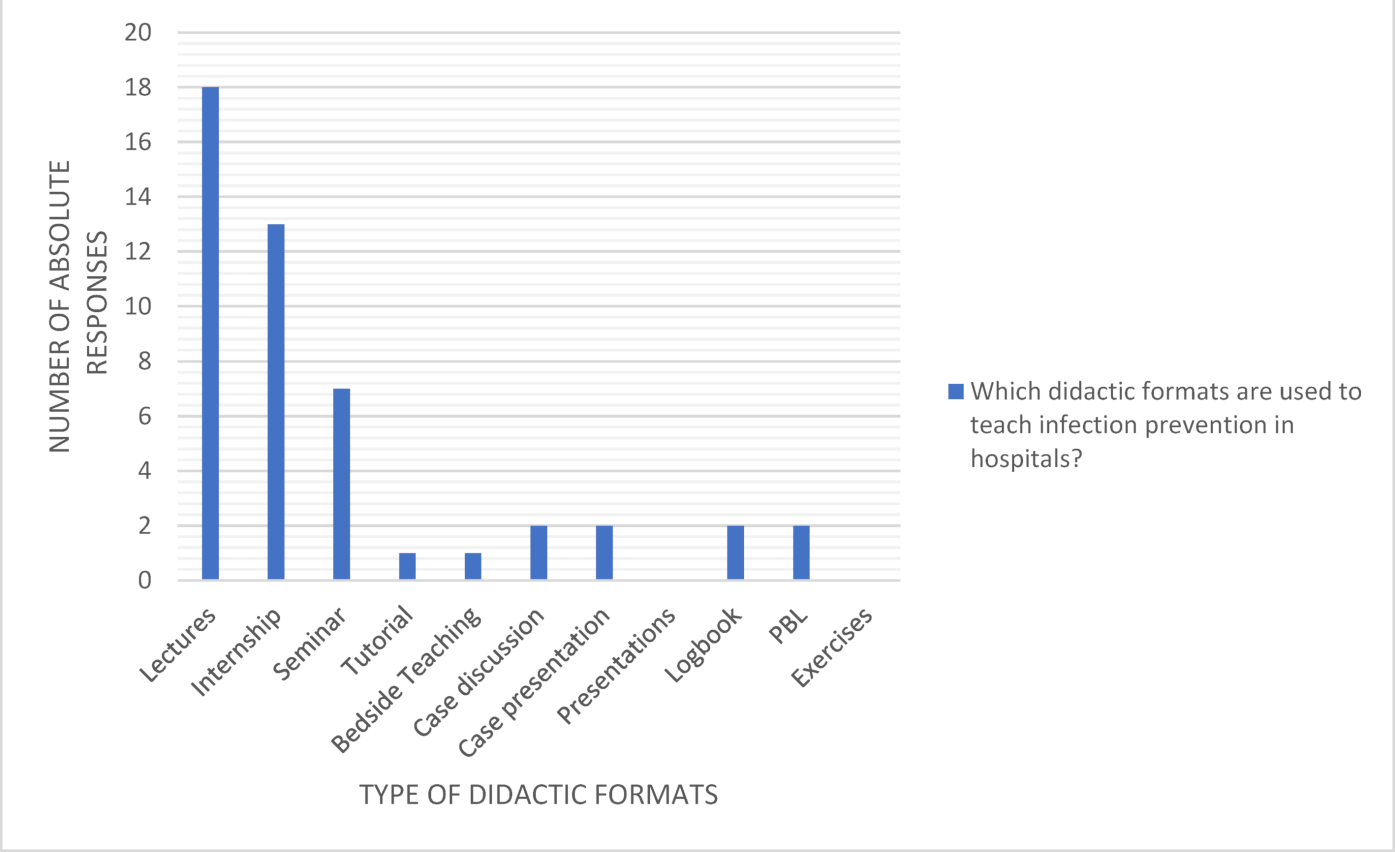
Didactic formats for teaching infection prevention in hospitals during medical studies (Multiple answers possible; answers from 19 locations; 48 answers in total)

Lectures (n=18), practical courses (laboratory practical training etc.) (n=13) and seminars (n=7) are used most frequently. Other formats are the exception. Seven locations use three different teaching methods, always lectures and usually also practical training and/or seminars. Six locations use two teaching methods, also always lectures and in four cases also practical training. Three locations only use one method, namely lectures. Exceptions are degree programs (n=2) in which a wide variety of different formats are used, including lectures and practical courses as well as problem-based learning, case presentations and discussions, logbooks and bedside teaching (see figure 4 (Fig. 4)).

**Figure 5:**
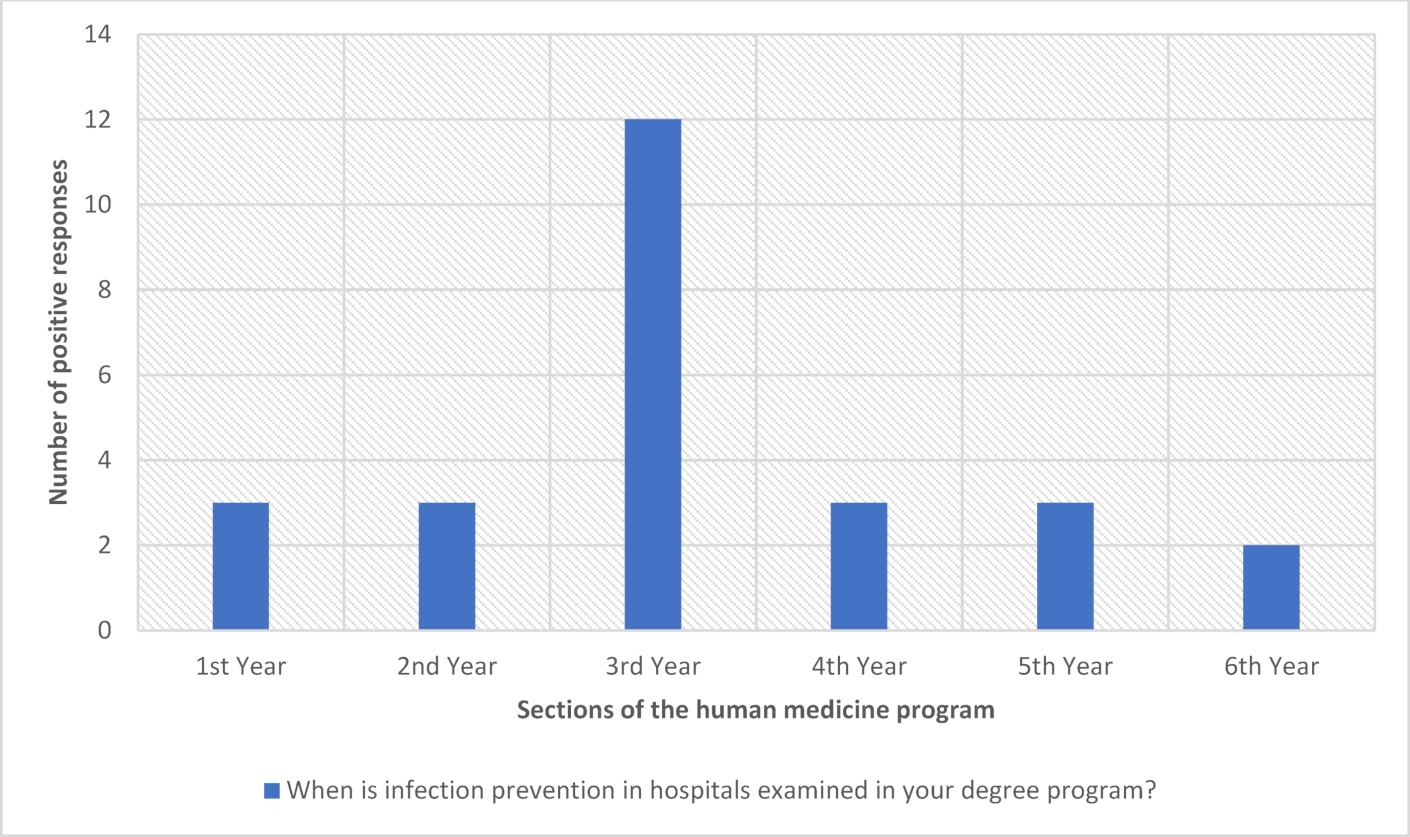
Timing of examinations on infection prevention in hospitals during medical studies (Multiple answers possible; answers from 19 locations; 26 answers in total)

Clinical placements (n=13), a self-selected rotation in the last year of 4 months (n=12), followed by internships (n=10) and work shadowing (n=2) are mentioned as contexts for clinical-practical instruction in infection prevention. At six locations, there are no offers to deepen the content of infection prevention in the hospital in a practical way. Three sites did not provide any information (see figure 5 (Fig. 5)). 

#### 3.2.5. What is taught?

In eleven out of 22 degree programs (50%), teachers use a superordinate model, a catalog of learning objectives or other instruments for curriculum planning when designing the content of teaching. Seven locations (32%) use the NKLM, two locations (9%) use the subject catalog of the Institute for Medical and Pharmaceutical Examination Questions (IMPP). Two study programs (9%) were not based on an overarching catalog of learning objectives. The respondents at six locations (27%) did not know the answer to this question and a further three locations (14%) did not provide any information. 

#### 3.2.6. Are gamification methods already in use?

Gamification is a familiar term for 11 (40%) of the total of 27 responses, both from the locations of the human medicine degree program and the specialist further nursing training in hygiene. Just over half (n=14; 52%) are not familiar with the term educational games. Irrespective of this, however, none of the surveyed study programs in human medicine or in the further nursing training of hygiene specialists use educational games in the context of teaching infection prevention in hospitals.

### 3.3. Organization and implementation of assessments of infection prevention audits in hospitals

#### 3.3.1. How are assessments carried out?

Of the 22 locations, 77% (n=17) assess content relevant to infection prevention in hospitals. To determine the pass mark, 77% (n=17) use standardized procedures, always with a fixed number of points or percentage (e.g. 60%). Two sites state that infection prevention in the hospital is not relevant for passing and is therefore assessed formatively. Three sites did not provide any information. Two degree programs also use formative assessment. In nine degree programs, formative knowledge tests are used in addition to examinations, for example as part of lectures. In one case, this form of knowledge tests replaces a pass-relevant examination.

#### 3.3.2. When are assessments carried out?

The most frequent examinations in infection prevention in hospitals take place in the 3^rd^ year of study (see figure 5 (Fig. 5)). At six medical locations (22%), examinations are held in at least two years of study and therefore several times. At nine locations (40%), examinations on this topic only take place once during the entire course of study. Two locations did not provide any information (see figure 5 (Fig. 5)). The distribution of examinations thus corresponds to the distribution of teaching (see figure 3 (Fig. 3)).

#### 3.3.3. Which assessment formats are used?

**Figure 6:**
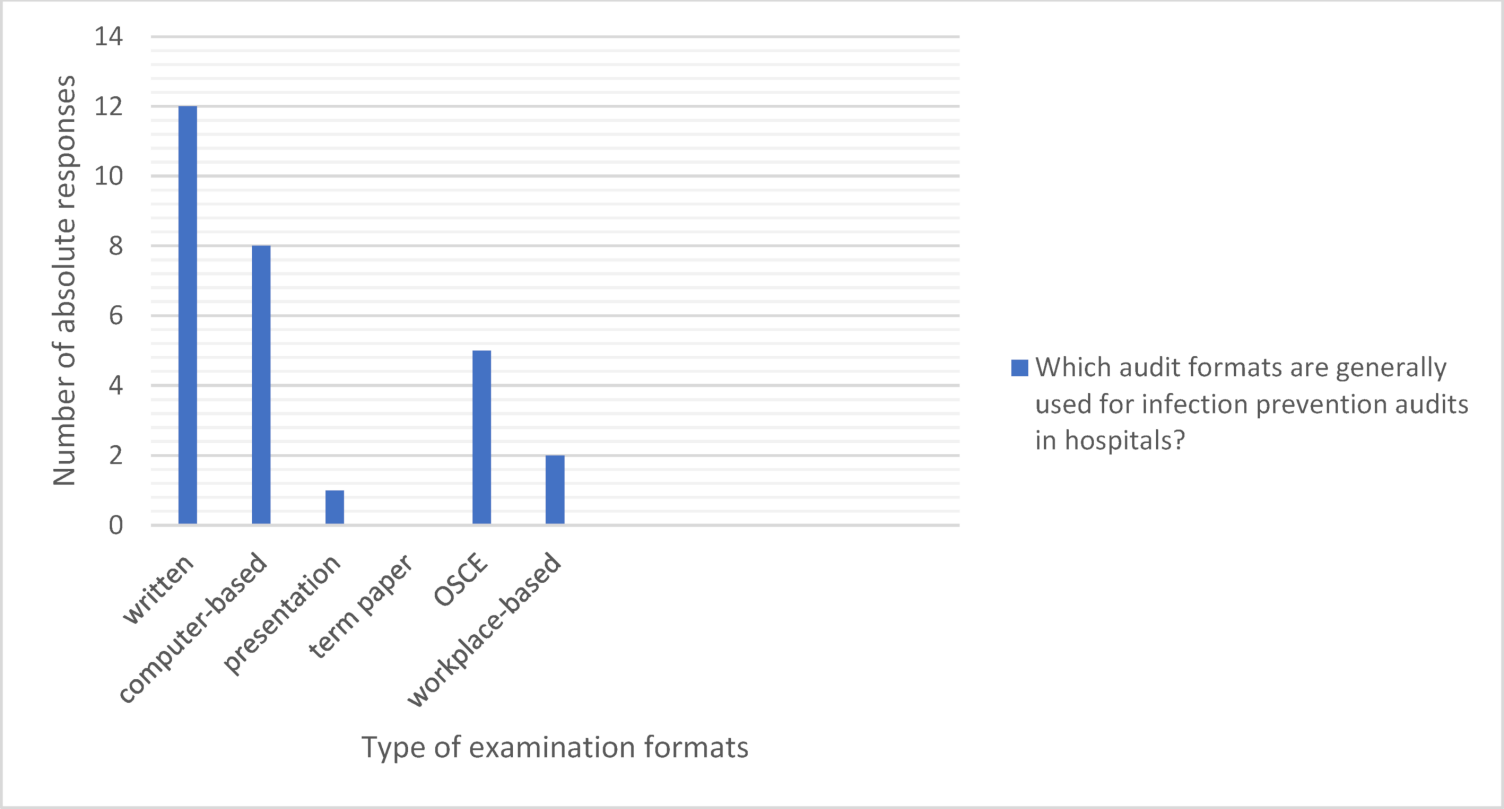
Examination formats for teaching infection prevention in hospitals during medical studies (Multiple answers possible; answers from 19 locations; 28 answers in total)

Written examinations (n=12) and computer-based examinations (n=8) are used most frequently in medical studies. At five locations, the content on infection prevention is also tested in the OSCE (see figure 6 (Fig. 6)).

#### 3.3.4. Who is responsible for the assessment?

Examiners for infection prevention in hospitals most frequently come from the field of microbiology, virology, and infection epidemiology (n=7), followed by (hospital) hygiene, although the professional qualifications are not defined more precisely here (n=5 each). There are also doctors from anesthesiology (n=1), infectiology (n=2) and biology (n=2) among the examiners. The question of whether the respective examiners also conduct the lessons preceding the examinations was answered in the affirmative by 7 locations (31.8%). At 9 locations (40.9%), the examiners do not hold the lessons themselves. For the remaining six locations, no answers were available or the answer was “don’t know”.

### 3.4. Most important summarized results for trainings for a specialist nurse in hygiene and infection prevention (FKHI)

Further training to become an FKHI takes place in recurring theory-practice blocks or in modules. The choice of teaching formats used shows a varied picture, led by presentations, (blocked) internships (n=5 each), case discussions, lectures, seminars, PBL and exercises (n=3 each), case presentations, and work shadowing (n=2 each). In terms of content, all locations are guided by the respective federal state-specific further training and examination regulations and the framework curriculum of the German Society for Hospital Hygiene as a concept development for training. Modules are completed with examinations, in addition to the final examination at the end of the training. The final examination is either oral or written. Examinations of modules include written examinations, presentations, and assignments. Computer-based examinations and OSCEs (n=1 each) are among the less frequently used examination formats. Examinations as part of workplace-based assessments are not used. Examiners are usually hygiene professionals themselves (n=5), followed by examiners from microbiology (n=3), and the public health department (n=1). 

## 4. Discussion and conclusions

### 4.1. Discussion 

The aim of the survey was to determine which learning objectives, teaching formats and examination formats are currently used in medical studies on the topic of infection prevention and to what extent. Overall, it is noticeable that the implementation of infection prevention teaching at the various medical schools varies considerably. This applies both to the scope of teaching, the placement in the degree program and the teaching formats. A certain overlap appears to be that teaching takes place primarily in the 3^rd^ year of study in the form of lectures and practical training and rarely comprises more than 20 hours in total. 

This seems particularly precarious in light of the fact that medical students can already have patient contact during the first two years of their studies (e.g. nursing internship; professional field exploration, examination courses on wards, internships in inpatient or outpatient settings) without receiving the necessary instruction and expertise in infection prevention at the earliest possible stage. This also harbors the potential for conflict with medical nursing staff, who are far better trained in hygiene and infection prevention [29]. This is different at US medical schools, with lectures predominantly in the first year of study [30]. Only a few locations offer teaching longitudinally, i.e. on a recurring basis. Case-based teaching or teaching in a clinical setting plays a subordinate role. Comprehensive catalogs of learning objectives have also not yet been established at all locations, which suggests a certain heterogeneity of teaching content. The NKLM appears to play a role in lesson design at only around a third of the locations. Unfortunately, information from a third of the locations is missing at this point.

In contrast, standardized state-specific training and examination regulations are used at the educational institutions providing trainings for a specialist nurse in hygiene and infection prevention. Even if, as expected, the number of hours spent there is not even remotely comparable to the number of hours spent on infection prevention in medical studies, the teaching formats, such as case discussions, case presentations, lectures, POL and exercises, appear to be more varied and also more practically oriented due to the structure in recurring theory-practice blocks or modules. 

In an international comparison, US medical schools seem to use significantly more teaching formats with regard to medical studies [30]. Future studies are needed to determine the extent to which a new licensing regulation and a nationally uniform catalog of learning objectives will lead to standardized teaching in the field of infection prevention in medical degree programs. 

So-called Entrusted Professional Activities (EPAs), which describe the minimum requirements for medical students with regard to professional medical practice, are already anchored in the IMPP's subject catalog for medicine [31]. These EPAs include, for example, “performing medical procedures in a patient-safe manner”, “carrying out prevention and early detection”, “identifying relevant risk factors” for a disease or determining vaccination status. In the future, this can form the basis for an integrated teaching approach for infection prevention in medical studies, which underpins the relevance of the topic in daily patient-related work in hospitals and is a more effective learning method than the usual ones. EPAs can also change medical education in infection prevention, as they are not time-fixed teaching units with variable learning outcomes but have a fixed learning outcome with variable time input [32]. In any case, the winters of 2022 and 2023 have shown that not only the COVID-19 pandemic, but also other waves of infection diseases will accompany our lives and work in the healthcare sector in the future and that the safe handling of preventive and protective measures, especially in terms of knowledge and practical skills, must be an integral part of medical studies. 

Taking into account the currently preferred assessment formats in infection prevention in hospitals – mostly written examinations – we critically question whether the development of factual knowledge in infection prevention alone can meet the requirements of everyday professional life in dealing with infection prevention issues as a clinical transfer performance, and why practical examination formats are not increasingly used. On the one hand, a sound theoretical knowledge of facts is indispensable; on the other hand, doctors are regularly faced with practical issues of infection prevention in almost all disciplines of direct patient care. Correctly performing hand disinfection, preparing infusions and blood products for transfusion, inserting an indwelling venous cannula, arterial punctures and disinfecting various examination objects after contact with patients – these are just a few specific examples of applications that can have a negative effect on the spread of infections in hospitals if not performed correctly. Orientation towards role models in both a negative and positive sense and the effects of risk perception also play a role [33].

The question therefore arises as to how knowledge and the acquisition of practical skills in the field of infection prevention can be promoted. From a learning theory perspective, case- and scenario-based teaching formats would be recommended that require and support the transfer of factual knowledge to prototypical clinical situations [34], [35], [36], [37]. In addition to case-based teaching formats that have been established for decades, such as problem-based or project-based learning, the use of serious games could also support the acquisition and consolidation of practical knowledge [38]. Although most participants in our study were aware of gamification, they were not or only rarely using it. However, serious games have already been successfully used and tested in medical training. Examples such as EMERGE by Raupach et. al. show positive effects in outcome-based studies, including on students’ knowledge retention [39]. A trend that can also be confirmed internationally [40], [41], [42].

### 4.2. Limitations of the study

In view of the increasing demand for interprofessional training of healthcare professionals [43] in response to the growing importance of interprofessional healthcare [9], [10], [44], [45], we also attempted in our survey to focus on the two healthcare professions that assume central responsibility for infection prevention in hospitals: medicine and nursing. Due to the number and diversity of educational institutions for healthcare and nursing, it was not possible to determine how infection prevention is currently represented in healthcare and nursing training as part of this project. In order to at least contrast – rather than compare – the medical studies with a nursing training course, we attempted to include the survey on the training for a specialist nurse in hygiene and infection prevention. Due to the low response rate, this could only be summarized, is not representative and just a small insight into the subject. After completing the study, we have to conclude that the structure of medical studies and a training for a specialist nurse in hygiene and infection prevention are too different for a meaningful mapping of both courses in a joint survey. Despite these limitations, we have decided to present some results, as we believe that it is worth considering a common objective in an interdisciplinary and interprofessional subject such as infection prevention, even though it is also clear that hygiene specialists are less likely to be users of infection prevention in healthcare facilities later on. Their role will be more of an advisory and controlling nature, while medical staff are primarily among the professional groups that apply it. It would make sense to develop common learning objectives and interprofessional teaching formats for medical studies and generalist nursing training in order to strengthen the interprofessional concept of infection prevention in hospitals in the interests of patient safety right from the training stage. 

The focus in developing the questionnaire was on questions about formats of teaching and examinations, as well as areas of responsibility in the implementation of instruction on infection prevention in hospitals. It would have been desirable to go into more detail about the content of infection prevention teaching, apart from surveying the catalogs of learning objectives, but this would have made the questionnaire significantly longer, which in turn would have been at the expense of the response rate. We therefore had to forego in-depth questions on further teaching content. 

Medical faculties from all parts of Germany took part in the online survey on medical studies, faculties with large and small student cohorts, with standard and model study programs. With a response rate of 55%, it can therefore be assumed with a certain degree of caution that the results are sufficiently meaningful, although a larger response rate would certainly have been desirable. There were challenges in contacting the relevant chairs and/or departments in advance, not least due to the heterogeneous distribution of responsibility for teaching infection prevention in hospitals. The involvement of those responsible for hygiene in the management of the COVID-19 pandemic also represented a significant time limitation for participation in our study. Even though we tried to cover as broad a spectrum of teaching as possible by surveying potential lecturers in hygiene, environmental medicine, microbiology and virology, those in charge of skills labs and the student representatives, it is possible that the topic of infection prevention in hospitals is also taught and examined in other subjects. For this reason, it is also important to mention that the survey cannot claim to cover the entire hospital infection prevention curriculum at all participating locations. At the same time, there is probably the greatest overlap with the subject of hygiene in terms of teaching infection prevention. It was not always possible to achieve sufficient selectivity in the questionnaire and in the answers to the individual questions. 

### 4.3. Outlook

Intensive engagement of medical schools with the NKLM and a mapping of their own learning objectives and content might provide greater clarity in future as to which content is taught and examined by which specialist disciplines and to what extent. This is closely bound up with the question of whether longitudinal teaching formats achieve a more sustainable learning effect or whether the knowledge should be better taught in blocks and in what number of hours in each case. Studies have already provided evidence of the advantages of integrated longitudinal clinical block placements [46], [47], [48] or longitudinal compared to block teaching of communication skills [49]. Future research needs to determine the extent to which these results can be transferred to the teaching of infection prevention. It would therefore be interesting to repeat the online survey in a few years’ time in order to record the expected progress in this area. 

The topic of interprofessional infection prevention is of central importance not only for the hospital sector, but also for the outpatient sector, the rehabilitation sector and the public healthcare sector, and basic principles of infection prevention can certainly be transferred from one sector to another. Nevertheless, the fields of application differ. It would be desirable to take more account of this in a future study than we were able to do with the present study. Subsequent surveys might also record the extent to which infection prevention content is taught and assessed in other medical-therapeutic professions in order to compare data. Ultimately, almost all medical-therapeutic professionals, be they doctors, nurses or physiotherapists, but also emergency paramedics, for example, have close contact with patients in a (pre-)clinical setting and are therefore highly relevant when it comes to achieving effective infection prevention.

### 4.4. Conclusions

The survey was primarily aimed at instruction and assessment relating to infection prevention in hospitals during medical studies. Due to the interprofessional implementation of infection prevention measures in hospitals, interprofessional training of medical students and nursing students would be an important contribution to anchoring common goals and standards in training. The already implemented standardization of learning objectives in further training to become a hygiene specialist as well as the intensive theory-practice relationship can be interesting indications of joint interprofessional teaching in this area. Similarly, more practically oriented, case-based and scenario-based teaching may support the acquisition of knowledge and practical skills in the field. We also see this as an important starting point for the implementation of gamification, in which knowledge can be deepened and practical skills acquired in a playful and interdisciplinary way. 

## Authors’ ORCIDs

- Paul-Dierk Tingelhoff: 0009-0008-1068-5441
- Claudia Kiessling: 0000-0003-4104-4854
- Bertram Otto: 0000-0002-8474-9283

## Acknowledgements

Our special thanks go to all those who participated in creating the survey and answering it. We would especially like to thank the members of the VHD, the bvmd and the Chairman of the Practical Skills Committee of the Society for Medical Education Dr. med. Kai Schnabel for their support, Birgit Wershofen, MScN, and OstR Michael Schweig for their constructive and critical feedback, and Christina Wagner for her support in translating the English version of the article.

## Funding

This work was supported by the Else Kröner-Fresenius-Stiftung (EKFS) under the funding codes 2021_EKKP.109 and 2022_EKKP.114.

## Competing interests

The authors declare that they have no competing interests. 

## Supplementary Material

Questionnaire used in the online survey
