# Supplementary material for: Infection prevention in medical education – results of a descriptive cross-sectional study in Germany
Source: GMS J Med Educ. 2024 Feb 15;41(1):Doc4. doi: 10.3205/zma001659 (PMC10946213; doi:10.3205/zma001659)
Supplement: Questionnaire used in the online survey [file JME-41-4-s-001.pdf]

Attachment 1: Questionnaire used in the online survey

Dear participants,

Thank you for your willingness to participate in the survey on teaching *infection prevention in hospitals. If you have* any questions, please contact us at any time (e-mail: Bertram.Otto@uni-wh.de).

This survey contains 28 questions.

Questions about the organization

In this section, we will ask you about the organizational background.

1. Faculty location or course location:

   ____

2. Name of the educational institution:

   ____

3. Name of the degree program or course

   ____

4. How many participants on average complete the study program or course per year (or in two semesters or several courses)?

   ____

The following questions are aimed at degree programs in human medicine:

5. Does your faculty have an independent chair for infection prevention or (hospital) hygiene? Please select only one of the following answers:
   - ☐ Yes
   - ☐ No
   - ☐ I don't know that
6. Does your faculty have an independent institute for infection prevention or (hospital) hygiene? Please select only one of the following answers:
   - ☐ Yes
   - ☐ No
   - ☐ I don't know that
7. Does your faculty have a professorship for infection prevention or (hospital) hygiene? Please select only one of the following answers:
   - ☐ Yes
   - ☐ No
   - ☐ I don't know that

8. Does your faculty have a department for infection prevention or (hospital) hygiene? Please select only one of the following answers:
   - ☐ Yes
   - ☐ No
   - ☐ I don't know that
9. If an infection prevention or (hospital) hygiene department exists, in which facility is it located? **(The condition for answering question 9 was a positive answer to question 8!)**

   ____

10. Where, if not at a chair/institute for infection prevention or (hospital) hygiene, is the specialist responsibility for the area of *infection prevention in hospitals* in teaching and examination in your faculty? Please select all applicable answers:
    - ☐ Microbiology, virology and infection epidemiology
    - ☐ Internal medicine and infectiology
    - ☐ Occupational medicine
    - ☐ Social medicine
    - ☐ Other, namely ____
    - ☐ I don't know that

Questions about curriculum and teaching

In this section we ask you for information about the curriculum for *infection prevention in hospitals* in your educational program.

11. Does your degree program/course teach *infection prevention in hospitals?* Please select only one of the following answers: (If "No", please skip questions 12-17!)
    - ☐ Yes
    - ☐ No
    - ☐ I don't know that
12. In which academic years is *infection prevention* taught *in the hospital?* Please select all applicable answers:
    - ☐ 1st year of study
    - ☐ 2nd academic year
    - ☐ 3rd academic year
    - ☐ 4th academic year
    - ☐ 5th academic year
    - ☐ 6th academic year
    - ☐ I don't know that
    - ☐ If your degree program consists of flexible modules or similar, please describe this briefly:

13. In which group sizes is *infection prevention* taught *in the hospital*? Please select all applicable answers:
    - ☐ Less than 4 participants (e.g.: examination courses on patients)
    - ☐ Less than 7 participants (e.g.: patient demonstrations)
    - ☐ Less than 16 participants (e.g. internships)
    - ☐ Less than 21 participants (e.g. seminars)
    - ☐ Less than 61 participants (e.g.: exercises)
    - ☐ Larger than 60 participants (e.g.: lecture)
    - ☐ I don't know that
    - ☐ Other: ____

14. Which didactic formats are used to teach *infection prevention in hospitals*? Please select all applicable answers:
    - ☐ Lecture
    - ☐ Internship
    - ☐ Seminar
    - ☐ Tutorials
    - ☐ Bedside teaching
    - ☐ Case discussions
    - ☐ Case presentations
    - ☐ Presentations
    - ☐ Logbook/Portfolio
    - ☐ Problem-oriented learning
    - ☐ Exercises
    - ☐ Other: if yes, which ______________________________

15. What opportunities for clinical-practical teaching in the hospital are used in your curriculum? Please select all applicable answers:
    - ☐ Work shadowing
    - ☐ Block internship
    - ☐ Clinical traineeship
    - ☐ Tertial in the practical year
    - ☐ I don't know that
    - ☐ Other: ____

16. From the students'/participants' point of view, what is the scope of the lessons on infection prevention? (teaching units of 45 minutes each): Please select all applicable answers:
    - ☐ Lecture: _____ UE
    - ☐ Seminar: _____ UE
    - ☐ Tutorials/exercises/internships :_____ UE
    - ☐ Other formats: _____ UE

17. Is the curriculum based on a model or catalog of learning objectives or similar (e.g. subject catalog, NKLM, standard textbooks, etc.)? Please select all applicable answers and write a comment (version of the subject catalog used, extent of use of the NKLM, etc.):
    - ☐ Yes, namely:____
    - ☐ No
    - ☐ I don't know that
18. If you do not use a catalog of learning objectives or similar, please briefly describe what the content of infection prevention in hospitals is based on in your curriculum:

We would be pleased if you would kindly provide us with your faculty's own catalog of learning objectives or the orientation you use via e-mail for the evaluation in the context of this study *(e-mail address provided*)

19. Do you know *Educational Games* as a teaching format? Please select only one of the following answers:
    - ☐ Yes
    - ☐ No
    - ☐ I don't know that
20. Have you already used *educational games* as a teaching format for teaching infection prevention in hospitals? Please select only one of the following answers:
    - ☐ Yes, namely:____
    - ☐ No
    - ☐ I don't know that

Questions about the examination system

Information on individual examination formats and the organization of the examinations

21. Is *infection prevention in hospitals* tested in your study program/course? Please select only one of the following answers: (If "No", please skip questions 22-26!)
    - ☐ Yes
    - ☐ No
    - ☐ I don't know that
22. If yes, in which academic years is *infection prevention in hospitals* examined? Please select all applicable answers:
    - ☐ 1st year of study
    - ☐ 2nd academic year
    - ☐ 3rd academic year
    - ☐ 4th academic year
    - ☐ 5th academic year
    - ☐ 6th academic year
    - ☐ I don't know that
    - ☐ If your degree program consists of flexible modules or similar, please describe this briefly:

23. Which examination formats are generally used (summative and formative) for *infection prevention* examinations *in hospitals*? Please select all applicable answers:
    - ☐ Paper-based written formats
    - ☐ Computer-based formats
    - ☐ Presentations / Lectures
    - ☐ Term papers
    - ☐ OSCEs
    - ☐ Workplace-Based Assessment
    - ☐ I don't know that
    - ☐ Other: ______________________________
24. If your degree program/course includes a **pass-relevant** examination on *infection prevention in hospitals*, how is the pass mark for the pass-relevant examinations or the examination determined? Please select all applicable answers:
    - ☐ *Infection prevention in the hospital* is tested **non-existentially**
    - ☐ Fixed number of points / percentage (e.g. 60 %)
    - ☐ I don't know that
    - ☐ Other method: ______________________________
25. Which subjects do the examiners come from? (e.g. Hygiene and Environmental Medicine, Microbiology, Virology and Infection Epidemiology, Internal Medicine and Infectiology, ...) Please write your answer here!
26. Do the examiners also conduct the lessons? Please select only one of the following answers:
    - ☐ Yes
    - ☐ Partial
    - ☐ No
    - ☐ I don't know that

    Please write a comment on your selection!
27. Does another form of knowledge assessment of infection prevention in hospitals take place in your curriculum (e.g. as part of lectures, online)?
    - ☐ Yes
    - ☐ No
    - ☐ I don't know that

    Please write a comment on your selection!

Comments and additions to the survey

28. Here is space for comments, suggestions and additions to the survey:
